# Clinical, societal and personal recovery in schizophrenia spectrum disorders across time: states and annual transitions

**DOI:** 10.1192/bjp.2021.48

**Published:** 2021-07

**Authors:** Stynke Castelein, Marieke E. Timmerman, Mark van der Gaag, Ellen Visser, Johan Arends, Agna A. Bartels-Velthuis, Richard Bruggeman, Frederike Jörg, Gerdina H.M. Pijnenborg, Henderikus Knegtering, Wim Veling, Alex Wunderink

**Affiliations:** 1 Lentis Psychiatric Institute, Lentis Research, The Netherlands; and Faculty of Behavioural and Social Sciences, University of Groningen, The Netherlands; 2 Faculty of Behavioural and Social Sciences, University of Groningen, The Netherlands; 3 Department of Psychosis Research, Parnassia Psychiatric Institute, The Netherlands; and Faculty of Behavioural and Movement Sciences, Department of Clinical Psychology, Amsterdam Public Mental Health Research Institute, Vrije Universiteit Amsterdam, The Netherlands; 4 Rob Giel Research Center, University Center for Psychiatry, University Medical Center Groningen, University of Groningen, The Netherlands

**Keywords:** Epidemiology, psychotic disorders, schizophrenia, social functioning, outcome studies

## Abstract

**Background:**

Recovery in schizophrenia is a complex process, involving clinical, societal and personal recovery. Until now, studies analysed these domains separately, without examining their mutual relations and changes over time.

**Aims:**

This study aimed to examine different states of recovery and transition rates between states.

**Method:**

The Pharmacotherapy Monitoring and Outcome Survey (2006–2017) yearly assesses patients with schizophrenia in the Northern Netherlands. Data from 2327 patients with one up to 11 yearly measurements on clinical, societal and personal recovery were jointly analysed with a mixture latent Markov model (MLMM).

**Results:**

The selected MLMM had four states that differed in degree and pattern of recovery outcomes. Patients in state 1 were least recovered on any domain (16% of measurements), and partly recovered in states 2 (25%; featured by negative symptoms) and 3 (21%; featured by positive symptoms). Patients in state 4 (38%) were most recovered, except for work, study and housekeeping. At the subsequent measurement, the probability of remaining in the same state was 77–89%, transitioning to a better state was 4–12% and transitioning to a worse state was 4–6%; no transitions occurred between states 1 and 4. Female gender, shorter illness duration and less schizophrenia were more prevalent in better states.

**Conclusions:**

Quite a high recovery rate was present among a substantial part of the measurements (38%, state 4), with a high probability (89%) of remaining in this state. Transition rates in the other states might increase to a more favourable state by focusing on adequate treatment of negative and positive symptoms and societal problems.

Recovery in mental health is a complex process influenced by multiple factors, including the nature and severity of psychiatric symptoms,^1,2^ social determinants such as social network and work,^2–4^ and one's own experience of recovery, reflecting hope, identity and meaning.^5^ In line with the notion that multiple factors are involved,^6–8^ the recovery process differs across individuals and time.^9^ In mental health, we have focused on clinical recovery, aiming to minimise clinical symptoms. However, societal recovery (i.e. regaining everyday functioning in work, social relationships and housing) also strongly influences the way patients restore their health. Also, patients have emphasised the importance of personal recovery, referring to living a meaningful life.^10^ Until now, studies have examined the course of recovery in patients with schizophrenia in the clinical and/or societal domain,^1,11,12^ but never in all three domains simultaneously.

It is common practice to reduce data to dichotomous variables on subdomains, or to use a total score to reflect overall functioning. Both approaches imply that information is lost about which domains pose the biggest difficulties for patients. To address the aforementioned conceptual and methodological challenges, this study aims to offer a rich description of prevalent courses of clinical, societal and personal recovery among people with schizophrenia spectrum disorders, using data from a large, naturalistic follow-up cohort with yearly assessments. We aim to reflect the natural variability of recovery in a clinically meaningful way by using different, clinically meaningful outcome measures of clinical, societal and personal recovery. Using a mixture latent Markov model (MLMM),^13^ different states of recovery and transition rates between states will be examined. Capturing the broad information in a single model allows for insight into general patterns in the recovery processes of patients with schizophrenia.

## Method

### Study design and participants

In the Pharmacotherapy Monitoring and Outcome Survey (PHAMOUS), patients with a schizophrenia spectrum disorder are followed for as long as they receive treatment in four mental healthcare institutes in the Northern Netherlands, with yearly assessments in many domains of their clinical and social performance. The nature of this cohort has been described in detail elsewhere.^14^ Patients fulfilling the DSM-IV criteria for schizophrenia, schizoaffective disorder or other psychotic disorders, and who are aged ≥18 years, are included in PHAMOUS. For the current study, we selected those patients with at least one assessment of all three recovery domains (not all three in the same year), from 2006 until 2017; other patients are denoted as lost to follow-up.

To cover the three recovery domains (clinical, societal and personal), we selected 12 relevant recovery measures. Further, we examined the relationship between recovery measures and the following sociodemographic and patient characteristics: age, gender, main diagnosis as reported in the medical file according to DSM IV-TR and DSM-5 (since 2013), age at onset of first psychotic episode, age at first mental healthcare contact and prescription of antipsychotics.

The authors assert that all procedures contributing to this work comply with the ethical standards of the relevant national and institutional committees on human experimentation and with the Helsinki Declaration of 1975, as revised in 2008. Ethical approval was granted by the Medical Ethics Review Board of the University Medical Center Groningen (approval number METc 2015/347). No informed consent of patients was needed since PHAMOUS is part of regular medical care. The data of this study was obtained from the administrative care systems of the participating institutions.

### Recovery outcomes used in the MLMM

#### Assessment of clinical recovery

Clinical (symptomatic) recovery was assessed with the consensus schizophrenia remission items of the Positive and Negative Syndrome Scale (PANSS-R), with a time criterion of at least 6 months.^15^ The following eight items were assessed: delusions (P1), conceptual disorganization (P2) and hallucinatory behaviour (P3), blunted affect (N1), passive/apathetic social withdrawal (N4) and lack of spontaneity and flow of conversation (N6), mannerisms and posturing (G5) and unusual thought content (G9). Scores were collected based on a semi-structured interview. For each item, we categorised the answers as absent/unclear (score 1–2), mild/moderate (score 3–4) and severe (score 5–7).

#### Assessment of societal recovery

Societal recovery was assessed with the three items of the Functional Recovery Tool, observing three areas of functioning in the past 6 months: daily living and self-care; work, study and housekeeping; and social contacts.^16,17^ These areas were rated on a three-point scale in a semi-structured interview: independent functioning (score 0), partially independent functioning (score 1) and dependent functioning (score 2; total score 0–6).

#### Assessment of personal recovery

Personal recovery was assessed via the Single-Item Happiness Question (SIQ) as a proxy for personal recovery (range 0–10).^18^ The SIQ measures current happiness in life. The Connectedness, Hope, Identity, Meaning and Empowerment (CHIME-) framework is the most cited framework for personal recovery in mental health.^5^ There is not yet a golden standard for assessing personal recovery.^19^ However, there is an overall agreement that personal recovery is ‘personal and subjective’, and can only be assessed by the patients themselves. Studies outside the context of schizophrenia research have commonly demonstrated associations between happiness and ‘CHIME-related aspects’ such as hope, optimism, resilience, meaning and positive social relationships.^18,20–22^ Also, associations have been found within schizophrenia research.^23^

### Data analyses

Percentages and mean scores were used to describe the sociodemographic and patient characteristics, using SPSS version 25 for Windows. From the patients screened for PHAMOUS in the period 2006–2017, we compared included and non-included patients on the difference in mean (for continuous data) or proportion (for dichotomous data) on these characteristics, using 95% confidence intervals.

An MLMM was used to capture the prevalent courses of recovery on the 12 recovery measures among patients with psychotic disorders.^13,24–26^ The main idea of an MLMM is that the observed outcomes at measurement *t* depend on only the (latent) state of an individual patient at measurement *t*; further, the evolution across successive measurements is captured by the transition probabilities, entailing the probabilities of transferring from any of the states at measurement *t* − 1 to any of the states at measurement *t*. The transition probabilities may differ between patients, as each patient is presumed to belong to one class, where different classes have different transition probabilities. Thus, an MLMM consists of states (a latent categorical variable whose values may differ for each patient at each measurement) and classes (a latent categorical variable whose values may differ for each patient).

In our MLMM, we analysed the 12 recovery variables jointly to identify the states and the transition probabilities from one state to another, yielding maximum likelihood estimates based on all available data. We determined the number of latent states and the number of classes based on the statistical Bayesian information criterion (BIC), with minimal relative sizes of the states (i.e. across all measurements of all patients) and classes (i.e. of the patients) of 10%, and meeting the interpretability criterion of states, thereby eliminating models with only minor differences between subsets of states and/or classes. The BIC is a widely accepted index for latent categorical variable models.^24^ Specifically, we fitted MLMM models with the number of latent states running from one to six, and the number of classes from one to three; these numbers would be increased if the model with lowest BIC would be associated with six states and/or three classes. We selected the final model as having the lowest BIC value for which the class and state sizes were >10%.

To further characterise the classes and states of the selected MLMM model, they were subsequently related to the sociodemographic and patient characteristics. To this end, we used univariate regressions of the states (and classes) on each characteristic, taking into account the uncertainty owing to the estimation of the state (and class) memberships.^25^ These were exploratory tested with the chi-squared test with *α* = 0.01 (in view of the large sample size), with Bonferroni-adjusted correction for multiple hypothesis testing. All MLMM analyses were performed with the syntax module of Latent GOLD version 6.0 for Windows (Statistical Innovations, Arlington, MA, USA; see https://www.statisticalinnovations.com/latent-gold-6-0/).^26^ The modelling decisions are described in Supplementary Appendix 1 available at https://doi.org/10.1192/bjp.2021.48. The syntax files are available in Supplementary Appendices 2 and 3.

For reasons of comparison to earlier literature and to demonstrate the comparability of the current sample to samples used in other studies,^15,17,27^ we will also include recovery rates divided into two categories (i.e. recovered/not recovered), as are most commonly presented in previous studies (see Supplementary Appendix 4). Of note, we do not use these classifications in our MLMM (see above for classification of items in MLMM). With regard to clinical recovery, the cut-off points of the Schizophrenia Remission Working Group were used: a score of ≤3 on each item indicating symptomatic recovery; a score of 4–7 indicating no symptomatic recovery.^15^ Also, the classification of Leucht and Lasser was used, with a score of ≤2 on each item classed as ‘in recovery’ and a score of 3–7 classed as ‘not in recovery’.^27^ In this classification, ‘mild symptoms’ (score of 3) do not belong to the category ‘in recovery’.

With regard to societal recovery, the classification of Swildens et al was used.^17^ ‘In recovery’ stands for scoring ‘independent’ (zero) on the three domains. As this is a fairly strict classification, different classifications were also calculated. On personal recovery, a score of >7 on the SIQ was chosen as being ‘in recovery’.

## Results

### Sociodemographic and clinical characteristics

In total, 5699 patients with psychotic disorders were screened in PHAMOUS from 2006 until 2017. Of these patients, 2327 patients met the selection criterion for the current study, with a mean number of 4.4 (s.d. 2.4) yearly measurements. Measurements ranged from 1 (*n* = 298, 12.8%) to 11 (*n* = 6, 0.3%) yearly measurements. Included patients had in total 10 343 yearly measurements in the study period. We had in total 9420 PANSS-R measurements (91.1%, 9420/10 434), 5679 Functional Recovery Tool measurements (54.9%, 5679/10 343) and 6520 SIQ measurements (63.0%, 6520/10 343).

When comparing included patients with non-included patients, the former were more often male, slightly younger in age (1 year) and had a longer duration of both illness and treatment (both about 1.5 years) (all significant at *P* < 0.05) (Table 1). Patients included in our model showed similar ‘dichotomised recovery rates’ compared with previous studies (Supplementary Appendix 4).^15,17,27^

**Table 1:** Sociodemographic and clinical characteristics of included and non-included patients in the Markov model

Characteristic	Included patients, *n* = 2327		Non-included patients,^a^ *n* = 3372		95% CI of sample difference
	Mean	s.d.	Mean	s.d.	
Age, years	41.1	11.3	42.0	13.3	−1.52 to −0.23
Duration of illness, years^b^	13.8	10.7	12.5	11.1	0.69–1.97
Duration of treatment, years^b^	14.4	10.7	12.9	11.4	0.81–2.04
Gender	*n*	%	*n*	%	95% CI of sample difference
Male	1528	66.4	2104	62.9	1.02–6.08
Ethnicity					
White	2001	86.3	2934	88.5	−4.00 to −0.046
Black	116	5.0	128	3.9	0.04–2.24
Asian	65	2.8	83	2.5	−0.56 to 1.16
Indian/Latin American	19	0.8	9	0.3	0.14–0.95
Turkish	24	1.0	23	0.7	−0.16 to 0.84
Moroccan	22	0.9	34	1.0	−0.60 to 0.45
Other	73	3.1	105	3.2	−0.95 to 0.91
DSM-IV/5 diagnosis^c^					
Schizophrenia (codes 295.10, 20, 30, 40, 60, 90)	1470	63.2	1917	56.9	3.74–8.90
Schizoaffective disorder (codes 295.70)	347	14.9	488	14.5	−1.43 to 2.31
Substance-induced psychotic disorder (codes 291.30, 50 and 292.11, 12)	25	1.1	59	1.7	−1.28 to −0.07
Other psychotic disorders (codes 297.10 and 298.80, 90)	485	20.8	908	26.9	−8.31 to −3.86

a. Non-included: in the whole data-set, they did not have data for at least one assessment of all three recovery domains (not all three in the same year).

b. Reference year is patients’ first study assessment.

c. Most recent medical file diagnosis (not necessarily reference year 2012).

### MLMM model

The BIC values of the MLMM models with two to six latent states and one to three number of classes are presented in Table 2. The model with the lowest BIC value and the class and state sizes >10% is the MLMM with five states and one class. However, since the MLMM with the second-lowest BIC, with four states and one class, appeared easier to interpret, this one was selected (that is, from four to five states the interpretation remained similar, with one state split into two, with minor differences between the two). The selected model assigns all patients to one class, indicating that patients are rather homogenous in their transition probabilities.

**Table 2:** BIC values for MLMM with number of states 2–6 and number of classes 1–3

	Number of classes		
Number of states	1	2	3
2	**149** **354**	***149*** ***266***	**149** **285**
3	**145** **717**	***145*** ***659***	**145** **683**
4	***143*** ***815***	**143** **842**	**143** **907**
5	***142*** ***670***	**142** **741**	**142** **863**
6	*141* *851*	141 986	142 192

Smallest BIC value per number of states is depicted in italics; BIC values of models with minimal class and state sizes >10% are indicated in bold. BIC, Bayesian information criterion; MLMM, mixture latent Markov model.

### The four states

**Fig. 1:**
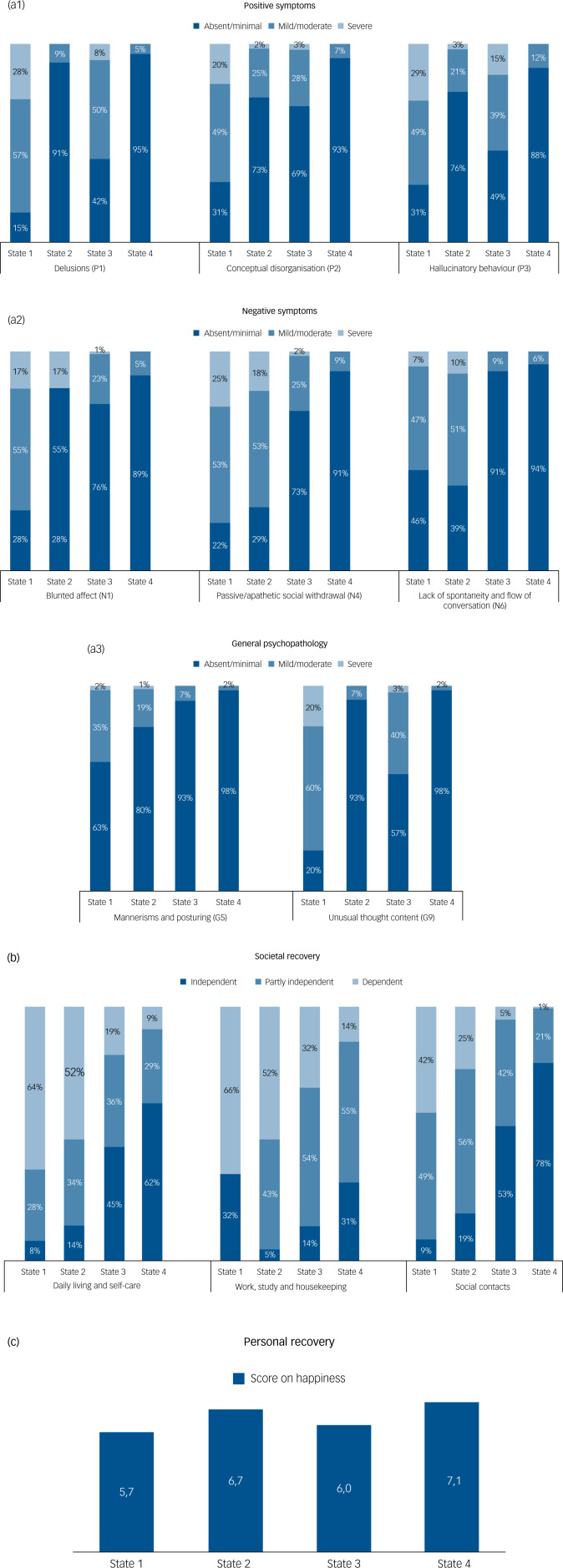
(a 1, 2, 3) Clinical recovery: percentages per state on positive symptoms, negative symptoms and general psychopathology (i.e. distribution per item per state). (b) Societal recovery: percentages per state on daily living and self-care; work, study and housekeeping; and social contacts (i.e. distribution per item per state). (c) Personal recovery: mean score on happiness per state. *Note*: P1, delusions; P2, conceptual disorganization P3, hallucinatory behaviour; N1, blunted affect; N4, passive/apathetic social withdrawal; N6, lack of spontaneity and flow of conversation; G5, mannerisms and posturing; G9, unusual thought content.

State 1 represents the least recovered outcome with regard to clinical, societal and personal recovery. Of all patients’ measurements, 16% belonged to this state. Figure 1 illustrates that on 11 out of 12 recovery outcome measures, patients in state 1 scored the highest impairment rate. In most measurements, patients scored mild/moderate and severe on clinical recovery measures (varying from 37 to 85%) and (partly) dependent on societal recovery (≥91%). The happiness score was low, at just sufficient (5.7). We therefore refer to this state as the ‘least recovered state’.

Of all patients’ measurements, 25% were in state 2. In state 2, positive symptoms were mostly absent, whereas these were present in approximately 70% in state 1 and about 30–60% in state 3. In contrast, negative symptoms were still frequently present, comparable with state 1 (about 60–70%). State 2 is also characterised by problems on the societal domain, but 12–17% less patients scored ‘severe’ on problems with daily living, work and social contact, compared with state 1. More happiness was reported compared with state 1 (6.7). Thus, state 2 seemed to have a better outcome on societal recovery compared with state 1, but worse societal outcome compared with states 3 and 4, and was furthermore characterised by predominantly negative symptoms. This state is referred to as the ‘partly recovered, negative symptom state’.

In state 3, 54–58% scored mild/moderate or severe on the positive symptoms ‘delusions’ and ‘hallucinatory behaviour’. Negative symptoms were less frequently reported compared with states 1 and 2 (>73% absent). Of all patients’ measurements, 21% were in this state. State 3 is characterised by a much better outcome on societal recovery compared with states 1 and 2, with 45–53% in this state functioning independently, except for the domain work. The happiness score was 5.9. A striking feature of this group is the presence of positive symptoms compared with states 2 and 4. We refer to this state as the ‘partly recovered, positive symptom state’.

State 4 is the best state on all recovery outcome measures (38% of all measurements). Although this state reported good outcomes on societal, clinical and personal recovery (dark blue boxes in Fig. 1), the domain work is still a major concern (55% partly independent, 14% dependent). In state 3, this phenomenon was also seen, i.e. good societal recovery outcome except for the outcome work. The happiness score was 7.1, the highest rating of the four groups. We refer to this state as the ‘most recovered state’.

### Age, gender and clinical differences per state

The four states differed statistically significantly from each other on gender, duration of illness and DSM-IV/5 diagnosis, but not on age and the use of antipsychotic medication (Table 3). Significant gender differences were detected between all states, except between the least recovered and partly recovered, negative symptom states. Men were relatively more present in the least recovered (79%) and partly recovered, negative symptom states (76%), compared with the other states (55–65%).

**Table 3:** Clinical characteristics per state (in proportions per state)

	State 1	State 2	State 3	State 4	Wald(0)	d.f.	*P*-value
	Least recovered	Partly recovered, negative symptoms	Partly recovered, positive symptoms	Most recovered			
Gender					62.17	3	<0.01
Male	0.79	0.76	0.65	0.55			
Female	0.20	0.23	0.34	0.43			
Missing	0.01	0.01	0.01	0.01			
Age, years					10.52	3	0.02
18–30	0.16	0.16	0.19	0.22			
30–40	0.25	0.25	0.25	0.26			
40–60	0.51	0.50	0.48	0.45			
≥60	0.07	0.08	0.07	0.06			
Missing	0.01	0.01	0.01	0.01			
Illness duration, years					26.28	3	<0.01
<5	0.15	0.19	0.21	0.26			
5–10	0.12	0.13	0.13	0.15			
10–20	0.27	0.27	0.26	0.25			
≥20	0.36	0.30	0.29	0.24			
Missing	0.10	0.11	0.11	0.10			
Antipsychotic medication					8.11	3	0.04
No	0.06	0.01	0.09	0.07			
Yes	0.94	0.99	0.91	0.93			
DSM-IV/5 diagnosis					77.45	3	<0.01
Schizophrenia (codes 295.10, 20, 30, 40, 60, 90)	0.69	0.76	0.57	0.42			
Schizoaffective disorder (codes 295.70)	0.12	0.11	0.13	0.13			
Substance-induced psychotic disorder (codes 291.30, 50 and 292.11, 12)	0.08	0.06	0.10	0.13			
Other psychotic disorders (codes 297.10 and 298.80, 90)	0.11	0.07	0.20	0.32			

Illness duration differed significantly between the least recovered and most recovered states. In the least recovered state, most patients had a duration of >20 years (36%). A shorter illness duration (<5 years) was found in 15% of those in the least recovered state, compared with 26% of those in the most recovered state.

More patients in the least recovered and partly recovered, negative symptom states suffered from schizophrenia, and fewer suffered from other psychotic disorders, compared with patients in the partly recovered, positive symptom and most recovered states.

### Transition rates between states

Table 4 presents the year-to-year transition rates between the states. The probability of remaining in the same state at the next measurement (i.e. year) ranged from 77–89% across the four states (displayed in bold). Transitions from the least recovered to the most recovered state and vice versa hardly occurred (0.31% and 0.49%, respectively). Higher transition rates were found between the other states, with a minimum of 4% and maximum of 12%. Also, the transition rate from the negative or positive symptom states to the most recovered state (both 10%) was twice as high compared with transition from the negative or positive symptom states to the least recovered state (4% and 6%, respectively).

**Table 4:** Transition rates between states in schizophrenia spectrum disorders

	State 1 at *t*	State 2	State 3	State 4
State at *t* − 1	Least recovered	Partly recovered, negative symptoms	Partly recovered, positive symptoms	Most recovered
State 1	**0.77**	0.12	0.11	0.00
State 2	0.04	**0.82**	0.04	0.10
State 3	0.06	0.04	**0.81**	0.10
State 4	0.00	0.05	0.05	**0.89**

Table should be read from left (row, referring to measurement *t* − 1) to right (column, referring to measurement *t*). Transition rates between the same states are displayed in bold.

## Discussion

The aim of this study was to identify and examine different recovery states in patients with psychotic disorders over time, and transition rates between states. Clinical, societal and personal recovery were analysed jointly. Based on the selected MLMM, four different recovery states in patients with schizophrenia and related psychotic disorders can be distinguished. In the next measurement (i.e. a year later), the probability of remaining in the state of the previous year is much higher (≥77%) than switching to a different state.

Our results give insight in the recovery process of patients with psychotic disorders with a longer duration of illness (about 13 years) by analysing 12 recovery outcome measures in one model with a follow-up period up to 11 years. To our knowledge, this is the first time these outcome measures were analysed jointly. About 40% of the patients are in the best state, which means that they are recovered on almost all domains. In addition, we found a high probability (89%) of remaining in this state. These findings are encouraging compared with the (separate) recovery rates of the 50 studies included in the meta-analysis by Jääskelainen et al, in which an overall recovery rate of 13.5% was found with a follow-up of 2 years.^11^

One might argue that patients in PHAMOUS have a better outcome, given that almost 40% are in the best state, and that the cohort is not representative for patients with schizophrenia and related psychotic disorders. To frame our findings, we have applied the classifications of other studies to our data. Oorschot et al reported a clinical recovery rate in schizophrenia of 40%;^28^ in our study, this was 51%. Swildens et al showed that 13.7% of the patients with severe mental illness were socially recovered,^17^ compared with 15.5% in our study. Also, baseline data support the representativeness of our data in severe mental illness.

An advantage of our MLMM model is that it provides insight into care needs per state. This means care may be directed specifically toward these care needs, which might increase the transition rate from one state to another. In the best state (state 4), the greatest challenge is to improve patients’ outcome on societal recovery, and more specifically, on the aspect work, study and housekeeping (about 70% have this specific care need), to achieve full recovery. In state 3, symptom scores of patients are relatively high on the positive symptoms hallucinations, delusions and unusual thought content. Although half of these patients function well on daily living and social contacts, gains could be made on work, study and housekeeping (86% have a need of care). In state 2, care should be directed primarily on negative symptoms, particularly blunted affect and social withdrawal. Given the limited number of interventions shown to be effective on negative symptoms,^29^ it remains important to develop new interventions. Severe issues on all three domains define state 1, and as such no specific guidance for intervention can be formulated. However, since the largest difference between states 1 and 2 is the severity of positive symptoms (fewer in state 2), this group may represent either persons who have yet to receive adequate medication management or a treatment-resistant group.

Next, it would be interesting to combine our data with the mental healthcare consumption of patients. Did patients get adequate and sufficient treatment for their care needs? As we did not include data about care consumption, this question remains unanswered, and should be reported as a suggestion for future research. It would also be interesting to collect data about patients’ antipsychotic medication use, such as dosage, oral or depot antipsychotic medication and medication adherence. However, our model offers the opportunity to begin a broader discussion about the degree to which mental healthcare needs are met in psychosis care.

In our model, we used happiness as a proxy for personal recovery. We think that the subjective question about happiness was important to include in the model as a counterpart of the more objective ‘clinical recovery’ and ‘societal recovery’ outcome measures. Although happiness in our study seems to be related to a patient's recovery state, we would suggest that future studies measure personal recovery with a multiple-item questionnaire covering the CHIME framework domains.^5^

A striking finding was that patients with mostly negative symptoms scored higher on happiness compared with patients with mostly positive symptoms, but experienced more societal problems. An explanation could be that patients with negative symptoms are less aware of their mental health condition and/or are receiving more psychosocial and rehabilitation-related support, having no acute psychotic problems, whereas patients with positive symptoms suffer from their hallucinations or delusions, leading to a high burden of illness.

Strengths of this MLMM include that the recovery states were calculated on outcomes of >2000 patients with a psychotic disorder, using longitudinal data with up to 11 years of follow-up. Furthermore, the use of this Markov model allowed us to analyse clinical, societal and personal recovery jointly, in contrast to other statistical models. We used data with multiple ordered categories, to allow for more fine-grained distinctions between the states of patients than dichotomous classifications of recovery.

As PHAMOUS is a naturalistic cohort study, patients lost to follow-up should be discussed as a limitation. We considered it a possibility that patients could have dropped out because they either recovered or suffered too much from symptoms to be able to participate in the annual screening. Therefore, we analysed sociodemographic and patient characteristics of both groups (patients included in the MLMM versus patients lost to follow-up). Although this led to statistically significant findings, the findings were not clinically relevant, as differences in illness duration, treatment duration and age were all about 1 year. Next, in both groups, men were more present than women (about 60%), and about 85% was Caucasian.

It should be noted that patients included in PHAMOUS represent a chronically ill group of patients with schizophrenia spectrum disorders. Thus, the results of the MLMM may not generalise to other patient groups (e.g. first episode). Another limitation is that the diagnoses might have been outdated, as we used the chart diagnosis from the medical file.

The last limitation is that PHAMOUS assesses patients’ outcomes yearly, indicating that changes within shorter periods could not be detected. However, most changes on recovery outcomes do take a considerable period of time, especially changes within the societal recovery domain and negative symptoms.^30^ We expect that we have captured the dynamics in recovery sufficiently, taking into account the illness duration of the included patients.

In summary, the four recovery states in our MLMM provide detailed information on care needs of patients with schizophrenia. Focusing on the specific needs per state might increase the transition rate to another state. Professionals could integrate the four recovery states in their care programme and treatment plans. Likewise, policy makers should implement these findings in their policy plans, and researchers could prioritise their agenda (e.g. placing a higher priority on societal recovery) as much as possible.

## Data Availability

Under the General Data Protection Regulation, our data is considered pseudonymised rather than anonymised, and is therefore still regarded as personal data. Given that participants have not given informed consent to have their personal data publicly shared, we are legally and ethically not allowed to publicly post our data-set. Data is therefore only available upon request to the Data Science Center of the Rob Giel Research Center (email: e.visser03@umcg.nl) or from the project leader, S.C. (email: s.castelein@rug.nl). Of note, syntax codes are shared in Supplementary Appendices 2 and 3.

## Supplementary material

For supplementary material accompanying this paper visit https://doi.org/10.1192/bjp.2021.48. click here to view supplementary material
