# Supplementary material for: Clinical, societal and personal recovery in schizophrenia spectrum disorders across time: states and annual transitions
Source: Br J Psychiatry. 2021 Jul;219(1):401–8. doi: 10.1192/bjp.2021.48 (PMC8529640; doi:10.1192/bjp.2021.48)
Supplement: Supplementary file 1 [file S0007125021000489sup001.zip › Appendix_4_Recovery_rates_per_type_no_changes_as.docx]

**Appendix 4.** Recovery rates per type (patients' first assessment in current study on this type of recovery)

|  | Cut-off points | N | Percent |
| --- | --- | --- | --- |
| Clinical recovery  (PANSS) | Scoring absent or minimal (≤2) on each item^a^ | 552 | 25.4 |
|  | Scoring absent, minimal or mild (≤3) on each item^b^ | 1147 | 52.8 |
| Societal recovery  (FR tool) | Scoring independent (0) on each domain^c^ | 313 | 13.7 |
|  | Scoring independent (0) on two domains, scoring partially independent (1) on one domain | 312 | 13.6 |
|  | Scoring partially independent (1) or higher on more domains | 1662 | 72.7 |
| Personal recovery  (SIQ) | Score 7 or higher | 594 | 25.6 |

^a^ In accordance with Leucht and Lasser (27); ^b^ In accordance with Andreasen et al. (15); ^c^ In accordance with Swildens et al. (17)
