# Supplementary material for: Clinical, societal and personal recovery in schizophrenia spectrum disorders across time: states and annual transitions
Source: Br J Psychiatry. 2021 Jul;219(1):401–8. doi: 10.1192/bjp.2021.48 (PMC8529640; doi:10.1192/bjp.2021.48)
Supplement: Supplementary file 1 [file S0007125021000489sup001.zip › Appendix_3_Latent_Gold_Syntax_predictors_no_changes_as.docx]

**Appendix 3** Latent Gold Syntax: predictors to classification

version = 5.1

infile 'Step1_C1_S4.sav'

model

title LM_C1_S4_age_group;

options

maxthreads=4;

algorithm

tolerance=1e-008 emtolerance=0,01 emiterations=250 nriterations=50 ;

startvalues

seed=0 sets=16 tolerance=1e-005 iterations=50;

bayes

categorical=1 variances=1 latent=0 poisson=1;

montecarlo

seed=0 sets=0 replicates=500 tolerance=1e-008;

quadrature nodes=10;

missing includeall;

step3 proportional ml;

output

parameters=effect betaopts=wl standarderrors=robust profile=posterior

probmeans=posterior;

variables

independent age_group;

latent State nominal posterior = (State#1 State#2 State#3 State#4);

psuid idnr;

equations

State <- 1 + age_group;

end model

model

title LM_C1_S4_gender;

options

maxthreads=4;

algorithm

tolerance=1e-008 emtolerance=0,01 emiterations=250 nriterations=50 ;

startvalues

seed=0 sets=16 tolerance=1e-005 iterations=50;

bayes

categorical=1 variances=1 latent=0 poisson=1;

montecarlo

seed=0 sets=0 replicates=500 tolerance=1e-008;

quadrature nodes=10;

missing includeall;

step3 proportional ml;

output

parameters=effect betaopts=wl standarderrors=robust profile=posterior

probmeans=posterior;

variables

independent gender;

latent State nominal posterior = (State#1 State#2 State#3 State#4);

psuid idnr;

equations

State <- 1 + gender;

end model

model

title LM_C1_S4_illness_duration_group;

options

maxthreads=4;

algorithm

tolerance=1e-008 emtolerance=0,01 emiterations=250 nriterations=50 ;

startvalues

seed=0 sets=16 tolerance=1e-005 iterations=50;

bayes

categorical=1 variances=1 latent=0 poisson=1;

montecarlo

seed=0 sets=0 replicates=500 tolerance=1e-008;

quadrature nodes=10;

missing includeall;

step3 proportional ml;

output

parameters=effect betaopts=wl standarderrors=robust profile=posterior

probmeans=posterior;

variables

independent illness_duration_group;

latent State nominal posterior = (State#1 State#2 State#3 State#4);

psuid idnr;

equations

State <- 1 + illness_duration_group;

end model

model

title LM_C1_S4_Antipsychotic;

options

maxthreads=4;

algorithm

tolerance=1e-008 emtolerance=0,01 emiterations=250 nriterations=50 ;

startvalues

seed=0 sets=16 tolerance=1e-005 iterations=50;

bayes

categorical=1 variances=1 latent=0 poisson=1;

montecarlo

seed=0 sets=0 replicates=500 tolerance=1e-008;

quadrature nodes=10;

missing includeall;

step3 proportional ml;

output

parameters=effect betaopts=wl standarderrors=robust profile=posterior

probmeans=posterior;

variables

independent Antipsychotic;

latent State nominal posterior = (State#1 State#2 State#3 State#4);

psuid idnr;

equations

State <- 1 + Antipsychotic;

end model

model

title LM_C1_S4_DSM_diagnosis;

options

maxthreads=4;

algorithm

tolerance=1e-008 emtolerance=0,01 emiterations=250 nriterations=50 ;

startvalues

seed=0 sets=16 tolerance=1e-005 iterations=50;

bayes

categorical=1 variances=1 latent=0 poisson=1;

montecarlo

seed=0 sets=0 replicates=500 tolerance=1e-008;

quadrature nodes=10;

missing includeall;

step3 proportional ml;

output

parameters=effect betaopts=wl standarderrors=robust profile=posterior

probmeans=posterior;

variables

independent DSM_diagnosis;

latent State nominal posterior = (State#1 State#2 State#3 State#4);

psuid idnr;

equations

State <- 1 + DSM_diagnosis;

end model
