# Supplementary material for: Clinical, societal and personal recovery in schizophrenia spectrum disorders across time: states and annual transitions
Source: Br J Psychiatry. 2021 Jul;219(1):401–8. doi: 10.1192/bjp.2021.48 (PMC8529640; doi:10.1192/bjp.2021.48)
Supplement: Supplementary file 1 [file S0007125021000489sup001.zip › Appendix_2_Latent_Gold_Syntax_MLMM_no_changes_as.docx]

**Appendix 2**  Latent Gold Syntax: MLMM

version = 5.1

//S2 = 2 latent states; C1 = 1 latent class

model

title LM_C1_S2;

options

maxthreads=4;

algorithm

tolerance=1e-008 emtolerance=0,01 emiterations=250 nriterations=50 ;

startvalues

seed=0 sets=16 tolerance=1e-005 iterations=50;

bayes

categorical=1 variances=1 latent=1 poisson=1;

montecarlo

seed=0 sets=0 replicates=500 tolerance=1e-008;

quadrature nodes=10;

missing includeall;

output

parameters=effect betaopts=wl standarderrors profile probmeans=posterior

bivariateresiduals estimatedvalues=model;

variables

caseid idnr;

dependent fr_living, fr_work, fr_contact, panss_p1, panss_p2, panss_p3,

panss_n1, panss_n4, panss_n6, panss_g5, panss_g9, happiness_1 continuous;

latent

Class nominal 1,

State dynamic nominal 2;

equations

Class <- 1; //For mixture latent class model - case specific latent variable.

State[=0] <- 1 | Class;

State <- (b1~tra) 1 | State[-1] Class;

fr_living <- 1 + State;

fr_work <- 1 + State;

fr_contact <- 1 + State;

panss_p1 <- 1 + State;

panss_p2 <- 1 + State;

panss_p3 <- 1 + State;

panss_n1 <- 1 + State;

panss_n4 <- 1 + State;

panss_n6 <- 1 + State;

panss_g5 <- 1 + State;

panss_g9 <- 1 + State;

happiness_1 <- 1 + State;

end model

//

model

title LM_C2_S2;

options

maxthreads=4;

algorithm

tolerance=1e-008 emtolerance=0,01 emiterations=250 nriterations=50 ;

startvalues

seed=0 sets=16 tolerance=1e-005 iterations=50;

bayes

categorical=1 variances=1 latent=1 poisson=1;

montecarlo

seed=0 sets=0 replicates=500 tolerance=1e-008;

quadrature nodes=10;

missing includeall;

output

parameters=effect betaopts=wl standarderrors profile probmeans=posterior

bivariateresiduals estimatedvalues=model;

variables

caseid idnr;

dependent fr_living, fr_work, fr_contact, panss_p1, panss_p2, panss_p3,

panss_n1, panss_n4, panss_n6, panss_g5, panss_g9, happiness_1 continuous;

latent

Class nominal 2,

State dynamic nominal 2;

equations

Class <- 1; //For mixture latent class model - case specific latent variable.

State[=0] <- 1 | Class;

State <- (b1~tra) 1 | State[-1] Class;

fr_living <- 1 + State;

fr_work <- 1 + State;

fr_contact <- 1 + State;

panss_p1 <- 1 + State;

panss_p2 <- 1 + State;

panss_p3 <- 1 + State;

panss_n1 <- 1 + State;

panss_n4 <- 1 + State;

panss_n6 <- 1 + State;

panss_g5 <- 1 + State;

panss_g9 <- 1 + State;

happiness_1 <- 1 + State;

end model

//

model

title LM_C3_S2;

options

maxthreads=4;

algorithm

tolerance=1e-008 emtolerance=0,01 emiterations=250 nriterations=50 ;

startvalues

seed=0 sets=16 tolerance=1e-005 iterations=50;

bayes

categorical=1 variances=1 latent=1 poisson=1;

montecarlo

seed=0 sets=0 replicates=500 tolerance=1e-008;

quadrature nodes=10;

missing includeall;

output

parameters=effect betaopts=wl standarderrors profile probmeans=posterior

bivariateresiduals estimatedvalues=model;

variables

caseid idnr;

dependent fr_living, fr_work, fr_contact, panss_p1, panss_p2, panss_p3,

panss_n1, panss_n4, panss_n6, panss_g5, panss_g9, happiness_1 continuous;

latent

Class nominal 3,

State dynamic nominal 2;

equations

Class <- 1; //For mixture latent class model - case specific latent variable.

State[=0] <- 1 | Class;

State <- (b1~tra) 1 | State[-1] Class;

fr_living <- 1 + State;

fr_work <- 1 + State;

fr_contact <- 1 + State;

panss_p1 <- 1 + State;

panss_p2 <- 1 + State;

panss_p3 <- 1 + State;

panss_n1 <- 1 + State;

panss_n4 <- 1 + State;

panss_n6 <- 1 + State;

panss_g5 <- 1 + State;

panss_g9 <- 1 + State;

happiness_1 <- 1 + State;

end model

//STATE3

model

title LM_C1_S3;

options

maxthreads=4;

algorithm

tolerance=1e-008 emtolerance=0,01 emiterations=250 nriterations=50 ;

startvalues

seed=0 sets=16 tolerance=1e-005 iterations=50;

bayes

categorical=1 variances=1 latent=1 poisson=1;

montecarlo

seed=0 sets=0 replicates=500 tolerance=1e-008;

quadrature nodes=10;

missing includeall;

output

parameters=effect betaopts=wl standarderrors profile probmeans=posterior

bivariateresiduals estimatedvalues=model;

variables

caseid idnr;

dependent fr_living, fr_work, fr_contact, panss_p1, panss_p2, panss_p3,

panss_n1, panss_n4, panss_n6, panss_g5, panss_g9, happiness_1 continuous;

latent

Class nominal 1,

State dynamic nominal 3;

equations

Class <- 1; //For mixture latent class model - case specific latent variable.

State[=0] <- 1 | Class;

State <- (b1~tra) 1 | State[-1] Class;

fr_living <- 1 + State;

fr_work <- 1 + State;

fr_contact <- 1 + State;

panss_p1 <- 1 + State;

panss_p2 <- 1 + State;

panss_p3 <- 1 + State;

panss_n1 <- 1 + State;

panss_n4 <- 1 + State;

panss_n6 <- 1 + State;

panss_g5 <- 1 + State;

panss_g9 <- 1 + State;

happiness_1 <- 1 + State;

end model

//

model

title LM_C2_S3;

options

maxthreads=4;

algorithm

tolerance=1e-008 emtolerance=0,01 emiterations=250 nriterations=50 ;

startvalues

seed=0 sets=16 tolerance=1e-005 iterations=50;

bayes

categorical=1 variances=1 latent=1 poisson=1;

montecarlo

seed=0 sets=0 replicates=500 tolerance=1e-008;

quadrature nodes=10;

missing includeall;

output

parameters=effect betaopts=wl standarderrors profile probmeans=posterior

bivariateresiduals estimatedvalues=model;

variables

caseid idnr;

dependent fr_living, fr_work, fr_contact, panss_p1, panss_p2, panss_p3,

panss_n1, panss_n4, panss_n6, panss_g5, panss_g9, happiness_1 continuous;

latent

Class nominal 2,

State dynamic nominal 3;

equations

Class <- 1; //For mixture latent class model - case specific latent variable.

State[=0] <- 1 | Class;

State <- (b1~tra) 1 | State[-1] Class;

fr_living <- 1 + State;

fr_work <- 1 + State;

fr_contact <- 1 + State;

panss_p1 <- 1 + State;

panss_p2 <- 1 + State;

panss_p3 <- 1 + State;

panss_n1 <- 1 + State;

panss_n4 <- 1 + State;

panss_n6 <- 1 + State;

panss_g5 <- 1 + State;

panss_g9 <- 1 + State;

happiness_1 <- 1 + State;

end model

//

model

title LM_C3_S3;

options

maxthreads=4;

algorithm

tolerance=1e-008 emtolerance=0,01 emiterations=250 nriterations=50 ;

startvalues

seed=0 sets=16 tolerance=1e-005 iterations=50;

bayes

categorical=1 variances=1 latent=1 poisson=1;

montecarlo

seed=0 sets=0 replicates=500 tolerance=1e-008;

quadrature nodes=10;

missing includeall;

output

parameters=effect betaopts=wl standarderrors profile probmeans=posterior

bivariateresiduals estimatedvalues=model;

variables

caseid idnr;

dependent fr_living, fr_work, fr_contact, panss_p1, panss_p2, panss_p3,

panss_n1, panss_n4, panss_n6, panss_g5, panss_g9, happiness_1 continuous;

latent

Class nominal 3,

State dynamic nominal 3;

equations

Class <- 1; //For mixture latent class model - case specific latent variable.

State[=0] <- 1 | Class;

State <- (b1~tra) 1 | State[-1] Class;

fr_living <- 1 + State;

fr_work <- 1 + State;

fr_contact <- 1 + State;

panss_p1 <- 1 + State;

panss_p2 <- 1 + State;

panss_p3 <- 1 + State;

panss_n1 <- 1 + State;

panss_n4 <- 1 + State;

panss_n6 <- 1 + State;

panss_g5 <- 1 + State;

panss_g9 <- 1 + State;

happiness_1 <- 1 + State;

end model

//STATE4

//

model

title LM_C1_S4;

options

maxthreads=4;

algorithm

tolerance=1e-008 emtolerance=0,01 emiterations=250 nriterations=50 ;

startvalues

seed=0 sets=16 tolerance=1e-005 iterations=50;

bayes

categorical=1 variances=1 latent=1 poisson=1;

montecarlo

seed=0 sets=0 replicates=500 tolerance=1e-008;

quadrature nodes=10;

missing includeall;

output

parameters=effect betaopts=wl standarderrors profile probmeans=posterior

bivariateresiduals estimatedvalues=model classification;

outfile 'X:\!files\ Step1_C1_S4.sav' classification;

variables

caseid idnr;

dependent fr_living, fr_work, fr_contact, panss_p1, panss_p2, panss_p3,

panss_n1, panss_n4, panss_n6, panss_g5, panss_g9, happiness_1 continuous;

latent

Class nominal 1,

State dynamic nominal 4;

equations

Class <- 1; //For mixture latent class model - case specific latent variable.

State[=0] <- 1 | Class;

State <- (b1~tra) 1 | State[-1] Class;

fr_living <- 1 + State;

fr_work <- 1 + State;

fr_contact <- 1 + State;

panss_p1 <- 1 + State;

panss_p2 <- 1 + State;

panss_p3 <- 1 + State;

panss_n1 <- 1 + State;

panss_n4 <- 1 + State;

panss_n6 <- 1 + State;

panss_g5 <- 1 + State;

panss_g9 <- 1 + State;

happiness_1 <- 1 + State;

end model

//

model

title LM_C2_S4;

options

maxthreads=4;

algorithm

tolerance=1e-008 emtolerance=0,01 emiterations=250 nriterations=50 ;

startvalues

seed=0 sets=16 tolerance=1e-005 iterations=50;

bayes

categorical=1 variances=1 latent=1 poisson=1;

montecarlo

seed=0 sets=0 replicates=500 tolerance=1e-008;

quadrature nodes=10;

missing includeall;

output

parameters=effect betaopts=wl standarderrors profile probmeans=posterior

bivariateresiduals estimatedvalues=model;

variables

caseid idnr;

dependent fr_living, fr_work, fr_contact, panss_p1, panss_p2, panss_p3,

panss_n1, panss_n4, panss_n6, panss_g5, panss_g9, happiness_1 continuous;

latent

Class nominal 2,

State dynamic nominal 4;

equations

Class <- 1; //For mixture latent class model - case specific latent variable.

State[=0] <- 1 | Class;

State <- (b1~tra) 1 | State[-1] Class;

fr_living <- 1 + State;

fr_work <- 1 + State;

fr_contact <- 1 + State;

panss_p1 <- 1 + State;

panss_p2 <- 1 + State;

panss_p3 <- 1 + State;

panss_n1 <- 1 + State;

panss_n4 <- 1 + State;

panss_n6 <- 1 + State;

panss_g5 <- 1 + State;

panss_g9 <- 1 + State;

happiness_1 <- 1 + State;

end model

//

model

title LM_C3_S4;

options

maxthreads=4;

algorithm

tolerance=1e-008 emtolerance=0,01 emiterations=250 nriterations=50 ;

startvalues

seed=0 sets=16 tolerance=1e-005 iterations=50;

bayes

categorical=1 variances=1 latent=1 poisson=1;

montecarlo

seed=0 sets=0 replicates=500 tolerance=1e-008;

quadrature nodes=10;

missing includeall;

output

parameters=effect betaopts=wl standarderrors profile probmeans=posterior

bivariateresiduals estimatedvalues=model;

variables

caseid idnr;

dependent fr_living, fr_work, fr_contact, panss_p1, panss_p2, panss_p3,

panss_n1, panss_n4, panss_n6, panss_g5, panss_g9, happiness_1 continuous;

latent

Class nominal 3,

State dynamic nominal 4;

equations

Class <- 1; //For mixture latent class model - case specific latent variable.

State[=0] <- 1 | Class;

State <- (b1~tra) 1 | State[-1] Class;

fr_living <- 1 + State;

fr_work <- 1 + State;

fr_contact <- 1 + State;

panss_p1 <- 1 + State;

panss_p2 <- 1 + State;

panss_p3 <- 1 + State;

panss_n1 <- 1 + State;

panss_n4 <- 1 + State;

panss_n6 <- 1 + State;

panss_g5 <- 1 + State;

panss_g9 <- 1 + State;

happiness_1 <- 1 + State;

end model

//STATE5

//

model

title LM_C1_S5;

options

maxthreads=4;

algorithm

tolerance=1e-008 emtolerance=0,01 emiterations=250 nriterations=50 ;

startvalues

seed=0 sets=16 tolerance=1e-005 iterations=50;

bayes

categorical=1 variances=1 latent=1 poisson=1;

montecarlo

seed=0 sets=0 replicates=500 tolerance=1e-008;

quadrature nodes=10;

missing includeall;

output

parameters=effect betaopts=wl standarderrors profile probmeans=posterior

bivariateresiduals estimatedvalues=model;

variables

caseid idnr;

dependent fr_living, fr_work, fr_contact, panss_p1, panss_p2, panss_p3,

panss_n1, panss_n4, panss_n6, panss_g5, panss_g9, happiness_1 continuous;

latent

Class nominal 1,

State dynamic nominal 5;

equations

Class <- 1; //For mixture latent class model - case specific latent variable.

State[=0] <- 1 | Class;

State <- (b1~tra) 1 | State[-1] Class;

fr_living <- 1 + State;

fr_work <- 1 + State;

fr_contact <- 1 + State;

panss_p1 <- 1 + State;

panss_p2 <- 1 + State;

panss_p3 <- 1 + State;

panss_n1 <- 1 + State;

panss_n4 <- 1 + State;

panss_n6 <- 1 + State;

panss_g5 <- 1 + State;

panss_g9 <- 1 + State;

happiness_1 <- 1 + State;

end model

//

model

title LM_C2_S5;

options

maxthreads=4;

algorithm

tolerance=1e-008 emtolerance=0,01 emiterations=250 nriterations=50 ;

startvalues

seed=0 sets=16 tolerance=1e-005 iterations=50;

bayes

categorical=1 variances=1 latent=1 poisson=1;

montecarlo

seed=0 sets=0 replicates=500 tolerance=1e-008;

quadrature nodes=10;

missing includeall;

output

parameters=effect betaopts=wl standarderrors profile probmeans=posterior

bivariateresiduals estimatedvalues=model;

variables

caseid idnr;

dependent fr_living, fr_work, fr_contact, panss_p1, panss_p2, panss_p3,

panss_n1, panss_n4, panss_n6, panss_g5, panss_g9, happiness_1 continuous;

latent

Class nominal 2,

State dynamic nominal 5;

equations

Class <- 1; //For mixture latent class model - case specific latent variable.

State[=0] <- 1 | Class;

State <- (b1~tra) 1 | State[-1] Class;

fr_living <- 1 + State;

fr_work <- 1 + State;

fr_contact <- 1 + State;

panss_p1 <- 1 + State;

panss_p2 <- 1 + State;

panss_p3 <- 1 + State;

panss_n1 <- 1 + State;

panss_n4 <- 1 + State;

panss_n6 <- 1 + State;

panss_g5 <- 1 + State;

panss_g9 <- 1 + State;

happiness_1 <- 1 + State;

end model

//

model

title LM_C3_S5;

options

maxthreads=4;

algorithm

tolerance=1e-008 emtolerance=0,01 emiterations=250 nriterations=50 ;

startvalues

seed=0 sets=16 tolerance=1e-005 iterations=50;

bayes

categorical=1 variances=1 latent=1 poisson=1;

montecarlo

seed=0 sets=0 replicates=500 tolerance=1e-008;

quadrature nodes=10;

missing includeall;

output

parameters=effect betaopts=wl standarderrors profile probmeans=posterior

bivariateresiduals estimatedvalues=model;

variables

caseid idnr;

dependent fr_living, fr_work, fr_contact, panss_p1, panss_p2, panss_p3,

panss_n1, panss_n4, panss_n6, panss_g5, panss_g9, happiness_1 continuous;

latent

Class nominal 3,

State dynamic nominal 5;

equations

Class <- 1; //For mixture latent class model - case specific latent variable.

State[=0] <- 1 | Class;

State <- (b1~tra) 1 | State[-1] Class;

fr_living <- 1 + State;

fr_work <- 1 + State;

fr_contact <- 1 + State;

panss_p1 <- 1 + State;

panss_p2 <- 1 + State;

panss_p3 <- 1 + State;

panss_n1 <- 1 + State;

panss_n4 <- 1 + State;

panss_n6 <- 1 + State;

panss_g5 <- 1 + State;

panss_g9 <- 1 + State;

happiness_1 <- 1 + State;

end model

//STATE6

//

model

title LM_C1_S6;

options

maxthreads=4;

algorithm

tolerance=1e-008 emtolerance=0,01 emiterations=250 nriterations=50 ;

startvalues

seed=0 sets=16 tolerance=1e-005 iterations=50;

bayes

categorical=1 variances=1 latent=1 poisson=1;

montecarlo

seed=0 sets=0 replicates=500 tolerance=1e-008;

quadrature nodes=10;

missing includeall;

output

parameters=effect betaopts=wl standarderrors profile probmeans=posterior

bivariateresiduals estimatedvalues=model;

variables

caseid idnr;

dependent fr_living, fr_work, fr_contact, panss_p1, panss_p2, panss_p3,

panss_n1, panss_n4, panss_n6, panss_g5, panss_g9, happiness_1 continuous;

latent

Class nominal 1,

State dynamic nominal 6;

equations

Class <- 1; //For mixture latent class model - case specific latent variable.

State[=0] <- 1 | Class;

State <- (b1~tra) 1 | State[-1] Class;

fr_living <- 1 + State;

fr_work <- 1 + State;

fr_contact <- 1 + State;

panss_p1 <- 1 + State;

panss_p2 <- 1 + State;

panss_p3 <- 1 + State;

panss_n1 <- 1 + State;

panss_n4 <- 1 + State;

panss_n6 <- 1 + State;

panss_g5 <- 1 + State;

panss_g9 <- 1 + State;

happiness_1 <- 1 + State;

end model

//

model

title LM_C2_S6;

options

maxthreads=4;

algorithm

tolerance=1e-008 emtolerance=0,01 emiterations=250 nriterations=50 ;

startvalues

seed=0 sets=16 tolerance=1e-005 iterations=50;

bayes

categorical=1 variances=1 latent=1 poisson=1;

montecarlo

seed=0 sets=0 replicates=500 tolerance=1e-008;

quadrature nodes=10;

missing includeall;

output

parameters=effect betaopts=wl standarderrors profile probmeans=posterior

bivariateresiduals estimatedvalues=model;

variables

caseid idnr;

dependent fr_living, fr_work, fr_contact, panss_p1, panss_p2, panss_p3,

panss_n1, panss_n4, panss_n6, panss_g5, panss_g9, happiness_1 continuous;

latent

Class nominal 2,

State dynamic nominal 6;

equations

Class <- 1; //For mixture latent class model - case specific latent variable.

State[=0] <- 1 | Class;

State <- (b1~tra) 1 | State[-1] Class;

fr_living <- 1 + State;

fr_work <- 1 + State;

fr_contact <- 1 + State;

panss_p1 <- 1 + State;

panss_p2 <- 1 + State;

panss_p3 <- 1 + State;

panss_n1 <- 1 + State;

panss_n4 <- 1 + State;

panss_n6 <- 1 + State;

panss_g5 <- 1 + State;

panss_g9 <- 1 + State;

happiness_1 <- 1 + State;

end model

//

model

title LM_C3_S6;

options

maxthreads=4;

algorithm

tolerance=1e-008 emtolerance=0,01 emiterations=250 nriterations=50 ;

startvalues

seed=0 sets=16 tolerance=1e-005 iterations=50;

bayes

categorical=1 variances=1 latent=1 poisson=1;

montecarlo

seed=0 sets=0 replicates=500 tolerance=1e-008;

quadrature nodes=10;

missing includeall;

output

parameters=effect betaopts=wl standarderrors profile probmeans=posterior

bivariateresiduals estimatedvalues=model;

variables

caseid idnr;

dependent fr_living, fr_work, fr_contact, panss_p1, panss_p2, panss_p3,

panss_n1, panss_n4, panss_n6, panss_g5, panss_g9, happiness_1 continuous;

latent

Class nominal 3,

State dynamic nominal 6;

equations

Class <- 1; //For mixture latent class model - case specific latent variable.

State[=0] <- 1 | Class;

State <- (b1~tra) 1 | State[-1] Class;

fr_living <- 1 + State;

fr_work <- 1 + State;

fr_contact <- 1 + State;

panss_p1 <- 1 + State;

panss_p2 <- 1 + State;

panss_p3 <- 1 + State;

panss_n1 <- 1 + State;

panss_n4 <- 1 + State;

panss_n6 <- 1 + State;

panss_g5 <- 1 + State;

panss_g9 <- 1 + State;

happiness_1 <- 1 + State;

end model
