# Supplementary material for: Clinical, societal and personal recovery in schizophrenia spectrum disorders across time: states and annual transitions
Source: Br J Psychiatry. 2021 Jul;219(1):401–8. doi: 10.1192/bjp.2021.48 (PMC8529640; doi:10.1192/bjp.2021.48)
Supplement: Supplementary file 1 [file S0007125021000489sup001.zip › Appendix_1_Modelling_decisions_of_the_MLMM_no_changes_as.docx]

**Appendix 1 Modelling decisions of the MLMM**

A mixture latent Markov model (MLMM) involves a time-varying discrete latent variable (i.e., states) and a time-constant latent variable (i.e., classes). The MLMM defines the distribution of the outcome variables. The formal definition can be found in Vermunt, Tran and Magidson^13^.

The assumptions are as follows:

a) the first order Markov assumption applies to the latent states; that is, conditional upon the class membership of individual *i*, the score on the latent state at time point *t* is only associated with the latent states at time point *t-1* and time point *t+1*, and not at other time points;

b) conditionally upon the class membership of individual *i* and their latent state at time point *t*, the observed responses on the outcome variables are independent of the latent states and the responses at the other time points; and

c) conditionally upon the class membership of individual *i* and their latent state at time point *t*, the observed responses on the outcome variables are mutually independent; this is known as the local independence assumption in latent class analysis.

Of the 12 outcome variables, ‘happiness’ was measured on a 10-point scale (range 1-10), and the others on three-point scales. For ‘happiness’, it was assumed that the distribution of the responses, conditional upon the class membership of individual *i* and their latent state at time point *t*, are normally distributed. For the other 11 outcome variables, it was assumed that their distribution conditional upon the class membership of individual *i* and their latent state at time point *t*, follow the adjacent-category logit model.

The MLMM was fitted using maximum likelihood estimation in LatentGOLD5.1^26^.

The MLMM involves a number of states and a number of classes. These numbers are unknown in practical applications, and hence need to be inferred from the data. A widely accepted index for latent categorical variable models^24^ is the use of the Bayesian Information Criterion (BIC) for model selection. The BIC is based on the model fit (i.e., log likelihood value) and the model complexity (i.e., numbers of states and classes). Among the range of models, the model with the lowest BIC has the most favourable balance between (good) model fit and (low) complexity. We further adopted as a criteria that a) the class and state sizes are larger than 10%, so as to avoid classes and states that pertain to rather small groups among the population, and b) that the solution was well-interpretable. We fitted all models with two to six states, in all combinations with one to three classes, thereby presuming that the model with the lowest BIC would have less than six states and three classes; if not, we would have extended the model complexity range by enlarging the number of states and classes.
